# Genome-wide DNA methylation of Munro’s microabscess reveals the epigenetic regulation in the pathogenesis of psoriasis

**DOI:** 10.3389/fimmu.2022.1057839

**Published:** 2022-12-08

**Authors:** Xiaoqing Xu, Yuxi Zhang, Zhaobing Pan, Xiaojing Zhang, Xiaonan Liu, Lili Tang, Xiaoguang Zhang, Fusheng Zhou, Hui Cheng

**Affiliations:** ^1^ Department of Dermatology, the First Affiliated Hospital, Anhui Medical University, Hefei, Anhui, China; ^2^ Institute of Dermatology, Anhui Medical University, Hefei, Anhui, China; ^3^ Key Laboratory of Dermatology (Anhui Medical University), Ministry of Education, Hefei, Anhui, China; ^4^ Department of Dermatology, The Second Hospital of Hebei Medical University, Shijiazhuang, Hebei, China; ^5^ Inflammation and Immune Mediated Diseases Laboratory of Anhui Province, Hefei, Anhui, China

**Keywords:** psoriasis, DNA methylation, Munro’s microabscess, pathology, neutrophil

## Abstract

**Introduction:**

Munro's microabscess is a typical pathological feature in the early psoriatic lesion, mainly characterized by the accumulation of neutrophils in the epidermis. DNA methylation microenvironment of Munro's microabscess and the crosstalk with transcription and its effect on neutrophils have not yet been revealed.

**Methods:**

Performed genome-wide DNA methylation analysis and further differential methylation analysis of psoriatic skin lesions with and without Munro's microabscess from two batch samples consisting of 114 former samples in the discovery stage and 21 newly-collected samples in the validation stage. Utilized GO, MEME, and other tools to conduct downstream analysis on differentially methylated sites (DMSs). Correlation analysis of methylation level and transcriptome data was also conducted.

**Results:**

We observed 647 overlapping DMSs associated with Munro's microabscess. Subsequently, GO pathway analysis revealed that DNA methylation might affect the physical properties associated with skin cells through focal adhesion and cellsubstrate junction and was likely to recruit neutrophils in the epidermis. Via the MEME tool, used to investigate the possible binding transcription factors (TFs) of 20 motifs around the 647 DMSs, it was found that DNA methylation regulated the binding of AP1 family members and the recruitment of neutrophils in the epidermis through the TGF-beta pathway and the TH17 pathway. Meanwhile, combined with our earlier transcriptome data, we found DNA methylation would regulate the expressions of CFDP, SIRT6, SMG6, TRAPPC9, HSD17B7, and KIAA0415, indicating these genes would potentially promote the process of Munro's microabscess.

**Discussion:**

In conclusion, DNA methylation may affect the course of psoriasis by regulating the progression of Munro's microabscess in psoriatic skin lesions.

## Introduction

Psoriasis is an immune-mediated, chronic, and inflammatory skin disease characterized by symmetrically well-defined erythema, covered with silvery scales, involving the elbows, knees, torso, and scalp (1, 2). Typical histopathological traits of psoriasis include hyperkeratosis with parakeratosis, immune cell infiltration, Munro’s microabscess, acanthosis thickening, vascular dilatation congestion, elongation of rete pegs, and granulosa thinning (1). In our previous studies, we explored the transcriptome (3) and chromatin accessibility patterns (4) in psoriatic lesions, and further data mining suggested that each pathological feature might be affected by both transcription and chromatin accessibilities, thus supporting the notion that these kinds of molecular changes would be involved in modulating the epidermal microenvironment (5). Munro’s microabscess is a characteristic pathological hallmark of early psoriasis, with inflammation encompassing polymorphonuclear leukocytes and forming within the skin’s epidermal layer, which can further trigger the following immune response in the surrounding microenvironment (6–8). Researchers have demonstrated that IL-1 signaling is vital for neutrophil recruitment and controlling chemokine expression in Munro’s microabscess formation in mouse psoriasiform imiquimod-induced skin inflammation (6). The pathogenesis of psoriasis is a complex cascade reaction. Therefore, it is necessary to investigate the emergence and regulation of the local microenvironment of Munro’s microabscess.

Genomic DNA methylation underlies the vast majority of biological inheritance. Healthy monozygotic twins of the same sex and age and nearly identical genomes exhibit indistinguishable methylomes at a young age and, over time, experience disparities and methylation differences through exposure to different environmental factors (9). Aberrant DNA methylation is associated with various inflammatory diseases and malignant tumors (10–12). Meanwhile, antitumor epigenetic drugs targeting DNA methylation have been developed and serve as a hotspot in the cancer treatment community (13).

As a systemic immune disorder, the occurrence and development of psoriasis involve both genetic and environmental factors (1). Furthermore, epigenetic modifications, such as DNA methylation, microRNA, chromatin remodeling, and other forms of non-DNA sequence changes, mediate the crosstalk between genetics and environmental factors (9, 10, 14). DNA methylation is an essential component of epigenetics and is associated with gene expression repression (15). All of the modifications in the genome would regulate the gene expression and further bring the corresponding phenotype. Earlier, we performed genome-wide DNA methylation for psoriatic peripheral blood mononuclear cell samples and psoriatic lesions, identified nine differentially methylated sites (DMSs) strongly related to psoriasis, and interrogated transcriptome to verify the expression of DMS-related genes (16). In the following research, we found differences in chromatin accessibility and gene expression in different pathological changes of psoriasis (5). At the same time, a study shows that DNA methylation analysis combined with gene expression displayed a discordant difference in monozygotic twins psoriasis (17). Another joint analysis study explored the DNA methylation patterns of local microenvironment and their functional regulation of gene expression among psoriatic lesions, non-lesions and healthy controls, which investigated skin biomarkers for psoriasis onset (18). Meanwhile, the cardinal pathological features of psoriasis are likely regulated by DNA methylation (19), but so far, the mechanism behind psoriasis pathological changes has not been fully studied by DNA methylation combined transcriptome. Therefore, the characteristics of methylation in the key pathological changes of psoriasis and its relationship with gene expression are worthy of further study.

To explore the methylation profiles of Munro’s microabscess, we first performed data mining of previously 114 psoriatic lesions with and without Munro’s microabscess. In order to confirm these results, we further collected 21 psoriatic tissue samples, including 4 with and 17 without microabscess, and assayed them using the same genome-wide methylation arrays. A total of 647 overlapped DMSs were subjected to further analysis, and we integrated and analyzed the DNA methylation and transcriptome. Overall, we explored the regulatory mechanism of methylation in Munro’s microabscess, a key pathological change in psoriatic skin lesions, and possibly a therapeutic target in psoriasis.

## Materials and methods

### Subjects

Skin tissue samples were collected from the Institute of Dermatology, Anhui Medical University. DNA methylation data from 114 samples and transcriptome data from 20 samples, and their detailed clinical features had been described previously (3, 16). The re-collected 21 DNA methylation samples follow the same procedures. All the patients in the study were included according to the following criteria: (I) more than one demarcated, erythematous, scaly lesion identified by at least two dermatologists; (II) the identification of each lesion by clinical histopathology; (III) before the skin biopsy, no systemic antipsoriatic therapy for two weeks; and (IV) before the biopsy, no topical antipsoriatic treatment within one week. Even if these standards are fully followed, it is difficult to avoid the long-term effects of previous drug use on skin lesions. Without other secondary lesions, skin biopsies were performed at the edge of the psoriatic area. All lesions were quickly frozen in liquid nitrogen immediately after removal. After 21 psoriatic lesions were collected, DNA was immediately isolated using the FlexiGene DNA Kit (Qiagen, Hilden, Germany).

### Histopathological analysis

The collected skin tissue was fixed, embedded in paraffin, and sliced, and then the overall histopathological abnormalities were evaluated by Hematoxylin–Eosin staining, including Munro’s microabscess, a characteristic feature of psoriasis. Finally, the differential methylation site analysis of samples with or without Munro’s microabscess was performed.

### Methylation analysis and identifying Munro’s microabscess-related differentially methylated sites

Genome-wide DNA methylation sequencing processing the DNA samples using the Infinium Human-Methylation450 array (Illumina 450K). The R software was used for bioinformatics analysis. Using the R package ChAMP (http://www.bioconductor.org/packages/release/bioc/vignettes/ChAMP/inst/doc/ChAMP.html), the locusby-locus nonparametric Wilcoxon ranked test was used to Munro’s microabscess-related differentially methylated sites (20). Differential methylation data were analyzed *via* Benjamini–Hochberg-adjusted t-tests with a P-value of 0.05. The detailed data processing steps can be found in our previous studies (16). The hypermethylation loci were defined as the methylation levels were higher in Munro’s microabscess group than that in without-Munro’s microabscess group, and vice versa, as hypomethylation.

### MEME analysis

We used the MEME suite (https://meme-suite.org/meme/doc/cite.html) to define motifs that can be bound by TFs of DNA sequences around the 20 bp nearby the hypermethylated and hypomethylated loci and further predict TFs.

### eFORGE analysis

The eFORGE v2.0 program (http://https://eforge.altiusinstitute.org/) is a web-based tool for analysis and interpretation *via* EWAS data. eFORGE identifies cell-type-specific regulatory components of a set of EWAS-recognized differentially methylated positions. For the 647 unique DMSs from the overlapping two sets, we assessed the enrichment and depletion of overlap with tissue-specific or cell-type-specific regulatory features, including DNase 1 hypersensitive sites (DHS), all 15 state chromatin marks, and all five H3 histone marks (H3K27me3, H3K4me1, H3K4me3, H3K36me3, H3K9me3) using eFORGE v2.0. The set of 647 DMSs was entered as input in eFORGE and tested separately for enrichment and depletion of overlap with each of the three putative functional elements (DHS, all 15 state chromatin marks, and all five H3 marks) compared to the respective data from consolidated ROADMAP epigenomics. –log10 binomial p-value was used to evaluate the significance. The Benjamini-Yekutieli (BY)-corrected false discovery rate was used to correct multiple independent tests. Enrichment or depletion was considered statistically significant at P < 0.05.

### GO and KEGG pathway analysis and transcribed data heatmap plotted

The data were all implemented and plotted using https://www.bioinformatics.com.cn, a free online platform for data analysis and visualization.

### Statistical analysis

Differential methylation statistical analyses were performed on the R (version 4.1.1) platform (https://www.R-project.org/). GraphPad Prism, Version 7.04 for Windows, was used to construct a volcano plot. The dmlTest and t-test were used to evaluate the significance. The correlation analysis was performed using SPSS. P < 0.05 was considered statistically significant.

## Results

### The genome-wide methylation profile of Munro’s microabscess in 114 tissue samples

**Figure 1:**
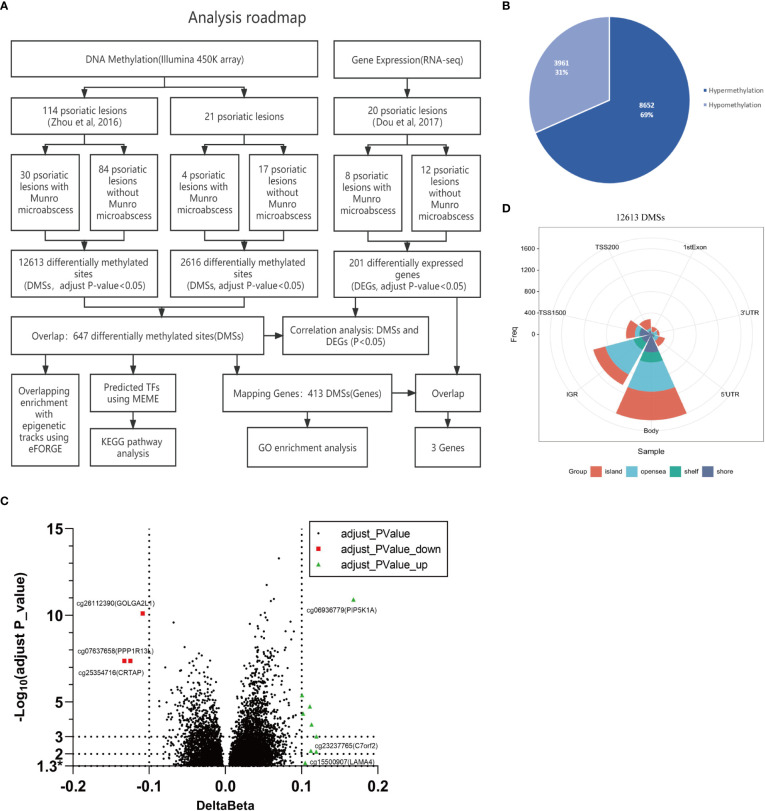
Idea map and differential methylation characteristics of 114 samples with and without Munro’s microabscess. **(A)** The whole analysis roadmap. **(B)** The number and proportion of hypermethylated and hypomethylated probes at 12,613 DMSs with or without Munro’s microabscess in 114 samples. **(C)** Volcano plot using -Log_10_(adjust P_value) and delta Beta at 12,613 DMSs.* stands adjust P_valve < 0.05. **(D)** Distribution of 12613 DMSs in CpG regions and on the genome.

We previously assayed genome-wide DNA methylation for psoriasis using the Infinium Human-Methylation450 array (Illumina 450K) (16). In that study, the 114 psoriatic skin samples could be subgrouped into 30 with and 84 without Munro’s microabscess. **Figure 1A** describes the main procedures in the current project. In brief, we extensively assayed the DNA methylation, gene expression, and their integration to reveal the main molecular features of Munro’s microabscess.

Firstly, we evaluated the genome-wide methylation differences between 30 samples with and 84 samples without Munro’s microabscess in psoriatic skin tissue. With a Benjamini–Hochberg-adjusted t-test P-value of 0.05, we found 12,613 DMSs, including 8,652 (69%) hypermethylation loci and 3,961 (31%) hypomethylation loci (Munro’s microabscess minus no Munro’s microabscess, **Figure 1B**). Additionally, with absolute delta beta difference (ΔBeta) > 0.05, we obtained 993 DMSs, with 833(84%) hypermethylation loci and 160(16%) hypomethylation loci. For the 15 most significantly hypomethylated DMSs, including cg26112390 (ΔBeta=-0.11, adjusted P-value= 7.77*10^-11^), cg25354716 (ΔBeta=-0.12, adjusted P-value=4.2*10^-8^), and cg07637658 (ΔBeta=-0.13, adjusted P-value=4.2*10^-8^), the mapping genes are *GOLGA2L1*, *CRTAP*, and *PPP1R13L*, and for the 15 most remarkably hypermethylated DMSs, including cg06936779 (ΔBeta=0.17, adjusted P-value=1.21*10^-11^), cg06662428 (ΔBeta=0.12, adjusted P-value=9.89*10^-4^), and cg23237765 (ΔBeta=0.12, adjusted P-value=7.38*10^-3^), the mapping genes are *PIP5K1A*, *PAOX*, and *C7orf20* (**Figure 1C**; **Supplementary Table 1**).

We explored the gene contexts of these 12,613 DMSs, 36% (1604/12,613) of which are located on the gene body, followed by IGR with 27% (1074/12,613) (**Figure 1D**). A similar methylation pattern can be observed for both hypermethylated and hypomethylated loci (**Supplementary Figure 1**, **Supplementary Table 1**). These results suggest that there may be some abnormal methylation of the targeted gene body and IGR to regulate the occurrence of Munro’s microabscess in psoriasis patients.

### The verification of DMSs in 21 fresh tissue samples

**Figure 2:**
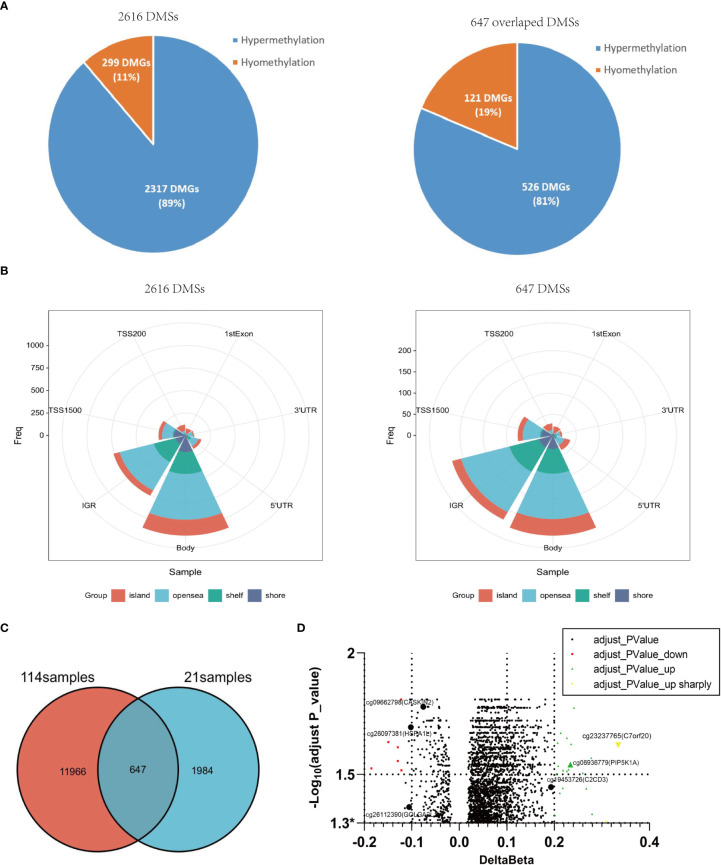
Differential methylation characteristics of 21 samples with and without Munro’s microabscess and overlapping with 114samples. **(A)**: The number, proportion, and distribution of 2,616 DMSs with or without Munro’s microabscess in 21 samples. **(B)** The overlapped DMSs between 114 samples and 21 samples. **(C)** The number, proportion, and distribution of 647 overlapped DMSs with or without Munro’s microabscess. **(D)** Volcano plot using -Log_10_(adjust P_value) and delta Beta at 2616 DMSs.* represents adjusted P-valve<0.05.

In order to confirm the above findings, we re-collected 21 psoriatic lesions, including 4 with and 17 without Munro’s microabscess, to profile genome-wide DNA methylation using the same methylation array (**Figure 1A**). In all samples, after filtering the ambiguous and duplicate probes, we detected 345,753 sites. With the same statistical strategy, we obtained 2,616 DMSs, containing 89% (2,317/2616) hypermethylated and 11% (299/2616) hypomethylated sites (**Figure 1A**, **Figure 2A**). Most of these DMSs were located on the gene body 43% (1,112/2,616) and IGR 30% (758/2,616) (**Figure 2A**). By overlapping the 12,613 DMSs generated from the 114 samples, we obtained 647 loci, including 526 (81%) hypermethylated and 121 (19%) hypomethylated loci (**Figure 1A**). The gene location context and hyper-/hypomethylation status of the 647 DMSs in the two sets are consistent (**Figure 2B, C**). Meanwhile, 5/15 of the most significant cases of hypomethylation are compatible with 114 psoriatic lesions’ data, with mapping genes including *GOLGA2L1*, *CASKIN2*, and *HSPA1L*; 12/15 are associated with hypermethylation, for which the mapping genes include *PIP5K1A*, *PAOX*, and *C7orf2* (**Figure 2D**; **Supplementary Table 1**).

To reveal the potential roles of these loci, we performed GO enrichment analysis on 413 genes that 647 DMSs mapped. Interestingly, 19 genes, such as *PIP5K1A*, *CAV3*, and *CLASP1*, were enriched in both focal adhesion and cell-substrate junctions (**Supplementary Table 2**). Furthermore, the methylation levels of 2/19 were hypomethylated, while 17 were hypermethylated (**Supplementary Table 3**). In addition, 13 of 19 sites were positioned within the gene body, and nine sites were in the open sea (**Supplementary Table 4**). These findings indicate that the 19 genes might chemotactically recruit leukocytes through focal adhesion and cell-substrate junctions, *via* changes in DNA methylation at the gene body, contributing to the formation of Munro’s microabscess.

### Predicted transcription factors on 647 overlapped DMSs

**Figure 3:**
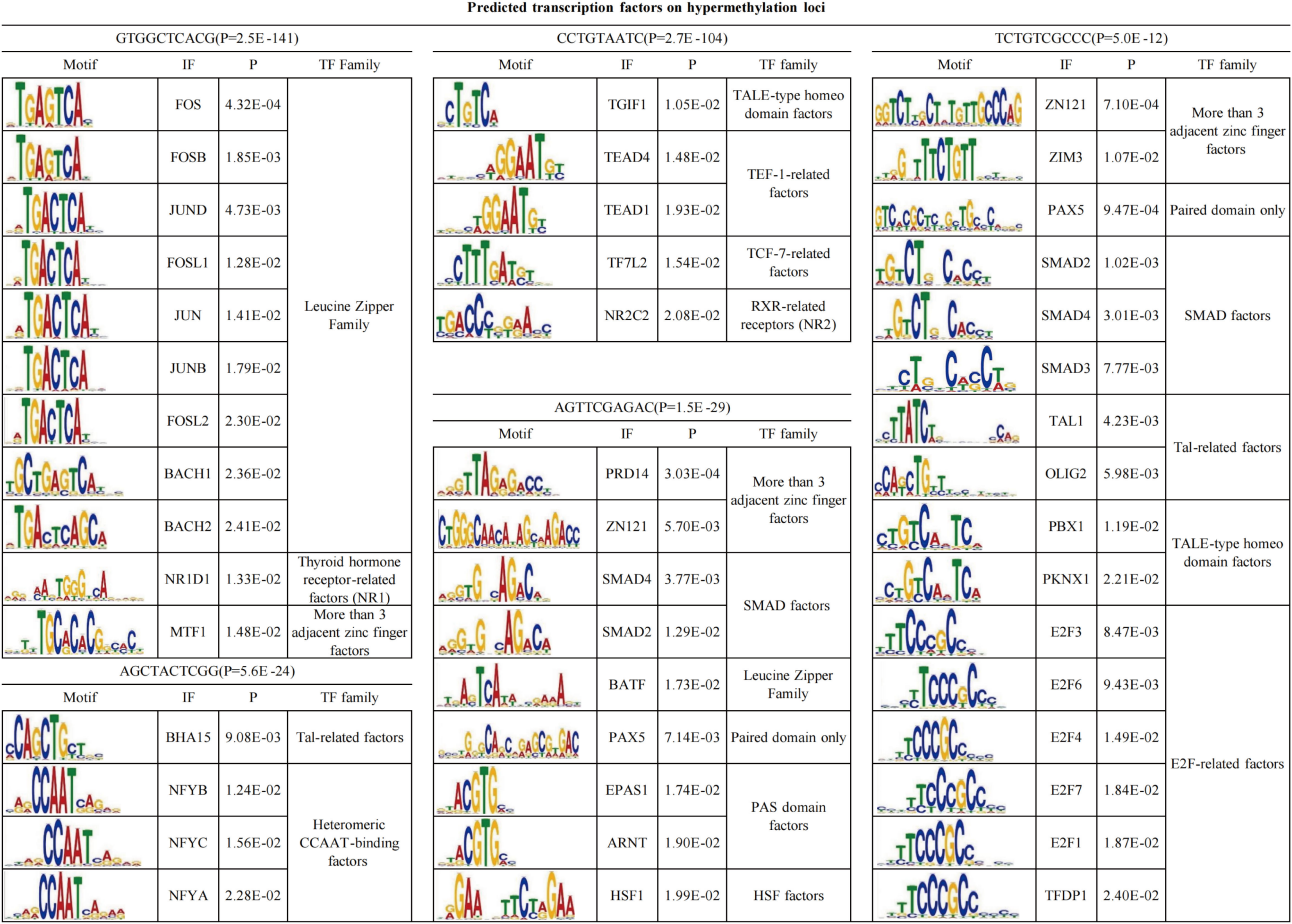
Predicted transcription factors on hypermethylation loci using MEME.

To further define whether DMSs would affect the binding of transcription factors (TFs), we performed Multiple Em for Motif Elicitation (MEME) motif analyses of DNA sequences around the 20 bp nearby the hypermethylated and hypomethylated loci, respectively (21). For the hypermethylated loci, five binding motifs were enriched around the GTGGCTCACG, CCTGTAATC, AGTTCGAGAC, AGCTACTCGG, TCTGTCGCCC (**Figure 3**). The most binding motif was GTGGCTCACG (P = 2.5*10^-141^). This motif can be bound by the leucine zipper family, covering *FOS*, *FOSB*, *JUND*, *FOSL1*, *JUN*, *JUNB*, *FOSL2*, *BACH1*, and *BACH2.* In addition, the second significant binding motif was CCTGTAATC (P = 2.7*10^-104^), which can be bound by *TGIF1*, *TEAD4*, *TEAD1*, *TF7L2*, and *NR2C2* (**Figure 3**). The other three motifs can be bound by a series of TFs, including *BHA15*, *PRD14, ZN121*, *SMAD4*, *BATF*, and others (**Figure 3**). A total of 47 TFs were identified for the five types of binding motifs. Subsequently, further KEGG pathway analysis showed that the 47 TFs were enriched in seven signaling pathways, such as human t-cell leukemia virus one injection, the cell cycle, the TGF-beta signaling pathway, and Th17 cell differentiation (P<0.0001, **Supplementary Figure 2**). The TGF-beta signaling pathway and Th17 cell differentiation are not only related to the pathogenesis of psoriasis but also the chemotaxis of neutrophils (22–25).

For hypomethylated sites, only one motif, TTYTCCGKYT, was observed. The motif can be bound by E2F-related factors, Ets-related factors, *MECP2*, *IRF*, *RUNX3*, and *OLIG2* (**Supplementary Figure 3**). *E2F*-related factors, including *TFDP1*, *E2F6*, *E2F7*, *E2F1*, *E2F4*, and *E2F3*, are crucial for the cell cycle, cell fate, and cell differentiation, including those of neutrophils (26, 27). In summary, DMSs would modulate the motif binding of several TFs and thus be involved in cellular biological processes during the accumulation of neutrophils in Munro’s microabscess.

### DNA methylation would regulate gene expression in Munro’s microabscess

**Figure 4:**
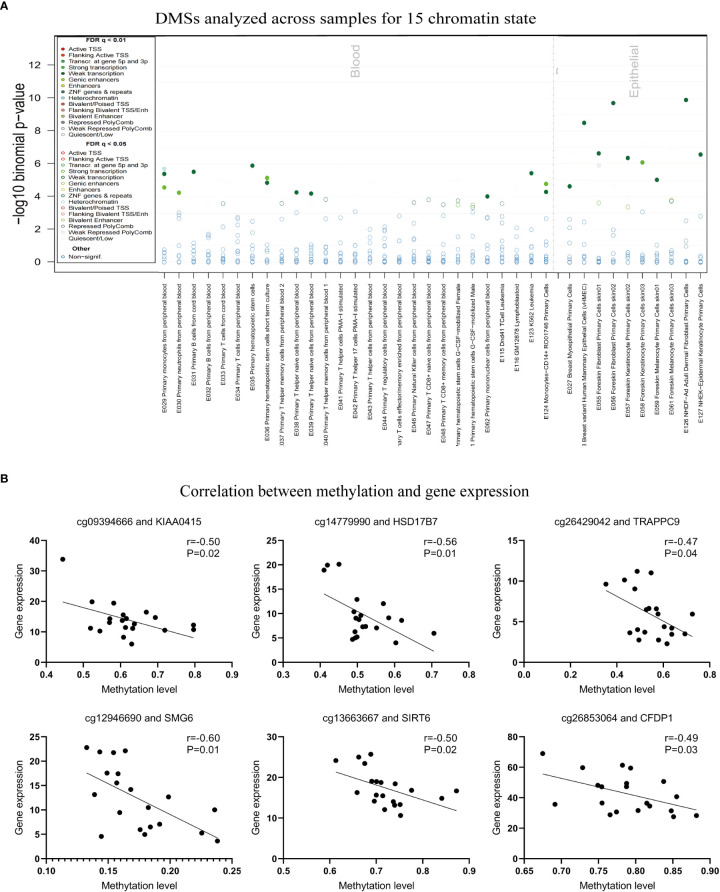
DNA methylation can be annotated by other types of epigenetic modifications *via* eForge, and the correlation between methylation and transcript expression in DMSs. **(A)** The Annotation of 15 chromatin states for 647 DMSs in blood and epithelial cells. **(B)** Correlation between methylation and gene expression.

The potential roles of DNA methylation can be annotated by other types of epigenetic modifications, such as DNaseI hotspots, five histone marks (H3K4me1, H3K4me3, H3K27me3, H3K9me3, and H3K36me3), and 15 chromatin states (**Figure 4A**). To functionally check whether the 647 DMSs could be annotated with other epigenetic tracks, we introduced the eFORGE algorithm, a useful tool to analyze and interpret cell-type-specific DNA methylation and epigenome data (28). The 1 kb surrounding the selected probes was searched as a proximity filter, and eFORGE utilizes data from the Roadmap Epigenomics, ENCODE, and BLUEPRINT projects, to calculate the enrichment scores and cell types functional annotation by comparing the overlap of DMSs and 1000 random probes with selected epigenetic markers, respectively. We found that some of the 647 DMSs could be annotated by histone modifications (**Supplementary Figure 4A**), DNaseI hotspots (**Supplementary Figure 4B**), and/or 15 chromatin states (**Figure 4A**). For example, the DMSs showed significant overlapping with H3K36me3 in monocytes from PBMC and primary T from cord blood, and with H3k9me3 in primary hematopoietic stem cells G-CSF-mobilization (**Supplementary Figure 4A**). The tracks of 15 chromatin states revealed a high enrichment of both blood and skin (**Figure 4A**). Moreover, the DMS signals are concentrated in various lymphocytes and neutrophils, potentially impacting gene transcription processes. At the same time, these DMSs were related to keratinocytes and other skin parenchyma cells (**Figure 4A**).

As a critical regulator of cell biology, DNA methylation is of great importance for regulating gene transcription (12). Based on our RNA-seq dataset previously generated from the same samples, we extracted the gene expression levels and compared the expression in patients in the presence and absence of Munro’s microabscess (3). Then, we obtained 201 differentially expressed genes (DEGs, t-test, p<0.05, **Figure 1A**). Based on these DEGs, 95% (20/21) of samples could be clearly separated, except for one sample without- been wrongly grouped into with-Munro’s microabscess (**Supplementary Figure 5A**). Additionally, the following genes showed the most remarkably increased expression in Munro’s microabscess: *COL5A2*, *CDCA7L*, and *DPP3* (**Supplementary Figure 5B**). In contrast, the most remarkably decreased expression genes in Munro’s microabscess were *DYNLRB2*, *C9orf103*, and *RFESD* (**Supplementary Figure 5C**). However, When 201 DEGs overlapped with 413 genes mapped by the 647 DMSs, we obtained only three genes, *PEMT*, *BAI2*, and *ACACA*, that were implicated in both methylation and expression analysis (**Supplementary Figure 5D**). Compared with the group without Munro’s microabscess, the *PEMT* gene expression was reduced, while the methylation level of the corresponding DMS was increased.

To further explore the role of these specific DMSs in gene expression, we correlated the level of methylation and gene expression level and found that cg26853064, cg13663667, cg12946690, cg26429042, cg14779990, and cg09394666 were negatively correlated with the expression of the mapping genes *CFDP1*, *SIRT6*, *SMG6*, *TRAPPC9*, *HSD17B7*, and *KIAA0415* (**Figure 4B**; **Supplementary Table 3**). In summary, these findings indicate that DNA methylation would potentially affect the formation of Munro’s microabscess and is probably closely related to neutrophils.

## Discussion

The onset of psoriasis is associated with genetic, immune, and environmental factors. The environment can interact with the genome through epigenetic modifications, including DNA methylation, affecting gene biological behavior and phenotype (15, 29). DNA methylation is involved in the pathogenesis of psoriasis, and the levels of 5-mC in psoriasis lesions are positively correlated with disease severity (15, 30, 31). Different pathological features of psoriasis tissue have distinguishing methylation patterns (19). Neutrophilic infiltrations that appear with Munro’s microabscess are hallmarks of the early psoriatic tissue reaction (1, 6). Our data show that Munro’s-related DMSs were more likely to appear in the gene body. DNA methylation at promoters is implicated in gene silencing, while the function of the gene body remains unclear. Studies have shown that Dnmt3b-dependent DNA methylation in the gene body prevents abnormal transcription initiation events and ensures mRNA transcription initiation fidelity (32, 33). Loss of transcription initiation fidelity may affect multiple biological processes, impairing molecular mechanisms such as gene expression regulation, miRNA targeting, and truncated protein production (33). A study showed abnormal RNA transcription in cancer is closely connected with gene body DNA methylation (34). To some extent, there is a positive correlation between DNA methylation and gene expression (35). Therefore, we speculate that Munro’s microabscess in early psoriasis may be linked with abnormal DNA methylation in the gene body.

Only 2,616 DMSs were obtained from 21 validation samples, far less than 12,613 DMSs in 114 samples. We reasoned this discrepancy would be due to insufficient sample size, as we saw only 4 Munro’s microabscess samples in the validation stage; this might dramatically decrease the statistical power to identify DMSs. And the functions of the genes matched by most significant hypomethylated DMSs are related to the composition of the intrinsic components of the cell, such as *GOLGA2L1* is involved in Golgi organization and spindle assembly, *CASKIN2* is a scaffold protein, *HSPA1L* lead to mitochondrial dysfunction (36, 37). In the meantime, *PIP5K1A* is the most significant hypermethylated DMSs and is related to the fundamental processes for cell migration, invasion, and metastasis in cancer (38). Those genes indicate that Munro’s microabscess skin cells may be abnormalities in cellular structure and function that lead to disease progression.

The GO enrichment analysis for genes corresponding to 647 DMSs indicated that 19 genes were enriched in two cellular components: focal adhesion and cell-substrate junctions. Interestingly, an abnormal condition regarding focal adhesion and cell-substrate junctions causes their reduced maturation and faster turnover and further affects the induction of cell adhesion, morphology, migration, and differentiation (39). We speculate that DNA methylation is mainly involved in the process of focal adhesion and cell-substrate function, and *via* the changes in biomechanics in skin cells, it favors neutrophil recruitment in the epidermis and further leads to the formation of Munro’s microabscess.

Then, the investigation of TFs that bind to 20 motifs around the hypermethylation probes produced five meaningful binding motifs. Interestingly, the leucine zipper family, including most AP1 family members, can bind to the most significant motif. A study using methylated binding microarrays showed that DNA methylation inhibited the binding of the leucine zipper family TFs (40). Deletion of AP1 family members, such as FOS, FOSB, and JUND, promotes inflammation characterized by neutrophil infiltration (41, 42). Accordingly, the most prominent motifs enriched at DNA hypermethylation sites in psoriatic Munro’s microabscess, favoring the promotion of the recruitment of neutrophils predominantly, can be bound by AP1-family TFs. Consequently, the results for all 47 TFs binding to hypermethylation loci showed seven signaling pathways. The human t-cell leukemia virus 1 injection pathway has the largest number of TFs enriched, and this suggests that there is a common partial mechanism or an unknown interaction between virus injection and Munro’s microabscess formed in psoriasis. The regulation of cell cycle signaling undergoes a transformation in the neutrophil population in the stress response status (43), as does the appearance of Munro’s microabscess. The osteoclast differentiation signaling pathway and IL-17 signaling pathway have a strong correlation with psoriatic arthritis (44–47). Interestingly, AP1 family members, FOS, FOSB, JUND, FOSL1, JUN, JUNB, and FOSL2 are enriched in osteoclast differentiation, while the first 5 TFs are also enriched in the IL-17 signaling pathway. In other words, the neutrophil aggregation in psoriasis with Munro’s microabscess may be combined with AP1 binding suppression, resulting from the DNA hypermethylation of AP1 binding sites. We hypothesize that DNA hypermethylation mediates the binding of the AP1 family to further contribute to Munro’s microabscess, and it may have a specific correlation with psoriatic arthritis.

At the same time, TGIF1, SMAD4, SMAD2, SMAD3, E2F4, and TFDP1 binding to the hypermethylation site motif is related to the TGF-beta signaling pathway, which can directly regulate the biological activities of neutrophils (23, 48). In addition, interestingly, the absence or inhibition of SMAD4 activity accounts for increased neutrophil infiltration (49). Therefore, hypermethylation of the SMAD-binding motif inhibits its binding to DNA and promotes neutrophil infiltration in the epidermis by regulating the TGF signaling pathway.

Various epigenetic modifications are crucial to regulating psoriasis pathogenesis—not only DNA methylation but also chromatin modification, histone modification, etc. (50). eFORGE is a web-based tool used to analyze and enrich overlapping with DNase I hypersensitive sites, five histone marks, and 15 chromatin states to determine the cell-type-specific regulatory component of a set of EWAS-identified differentially methylated positions (51). We performed eFORGE to overlap the 1 kb region around 647 DMSs and found some remarkable functional enrichment related to the chromatin state and histone modification. This suggests that Munro’s microabscess has an altered chromatin status. The signal of histone modification and chromatin state enrichment showed the functional enrichment of blood neutrophil-related signals, suggesting that neutrophils play a vital role in the onset of Munro’s microabscess. Histone modification also indicates the blood’s enrichment of monocytes and T-cell signals. Therefore, we speculate that interactions between various inflammatory cells may exist during Munro’s microabscess; there may also be a repetitive methylation profile in activating these inflammatory cells.

Interestingly, by comparing the transcriptome data from the same batch of samples with the 647 DMSs for GO pathway analysis, it was found that the enriched pathways, focal adhesion, and cell-substrate junction signaling pathway were consistent with the 647 DMSs. This shows the reliability of our data. Meanwhile, correlation analysis between DNA methylation levels and gene expression showed that six probes and their mapping genes’ expression have a meaningful negative correlation. It is reported that the knockdown of SIRT6 refreshes the corresponding recruitment of macrophages and neutrophils (52). A study found that circ-SMG6 may deteriorate myocardial I/R injury by promoting neutrophil recruitment (53). Hypermethylation levels of the cg26853064 and cg12946660 probes matched to *SIRT6* and *SMG6*, repressed gene expression, and were beneficial for neutrophil recruitment. The gene function of *HSD17B7* is controlled by altering epigenetic gene silencing in reverting cholesterol auxotrophy (54). TRAPPC9-related CPG methylation correlates with breastfeeding and early-life growth trajectories (55). The area near *KIAA0415* is differentially methylated in the orofacial cleft (56). However, there is not enough evidence for *CFDP1*, *TRAPPC9*, *HSD17B7*, and *KIAA0415*’s effects on psoriasis and neutrophils. Based on the available data, we can only speculate that the DNA methylation of these genes affects their expressionlevels and may regulate psoriatic Munro’s microabscess development.

In conclusion, this study defines a unique pattern of DNA methylation in the development of psoriatic Munro’s microabscess, characterized by changes in gene body methylation. It thus provides insight into possible DNA methylation within the local microenvironment of skin lesions in psoriasis onset. We also explored the possibility that the hypermethylation of AP1 family members and other transcription factor binding regions affects their binding on neutrophils through TGF-beta, TL-17, and other signaling pathways, thereby leading to the occurrence of Munro’s microabscess. The effects of other epigenetic modifications on 647 DMSs were excluded. At the same time, combined with the transcriptome data of psoriatic skin lesions from the same samples, it was explained that changes in methylation levels further regulate gene expression to affect the occurrence of Munro’s microabscess. The epigenetic changes in the local microenvironment of psoriasis skin lesions affect related signaling pathways to regulate the biological behavior of corresponding cells and the expression of associated genes, to then influence the occurrence and development of psoriasis pathology, and these results provide new insights into local, specific treatment for psoriasis.

## Data availability statement

The original contributions presented in the study are included in the article/**Supplementary Material**. Further inquiries can be directed to the corresponding authors.

## Ethics statement

The studies involving human participants were reviewed and approved by The Institutional Review Board and local Ethics Committee of the First Affiliated Hospital of Anhui Medical University. The patients/participants provided their written informed consent to participate in this study.

## Author contributions

HC, FZ, XX, YZ, and XGZ, carried out the design and bioinformatics analysis of this study. Samples were collected by ZP, XJZ, XL, and LT. XX and YZ writing the original draft preparation. HC, FZ, and XGZ reviewed and revised this manuscript. All authors have read and approved the final version of the manuscript. All authors contributed to the article and approved the submitted version.
