# Supplementary material for: Genome-wide DNA methylation of Munro’s microabscess reveals the epigenetic regulation in the pathogenesis of psoriasis
Source: Front Immunol. 2022 Dec 8;13:1057839. doi: 10.3389/fimmu.2022.1057839 (PMC9773074; doi:10.3389/fimmu.2022.1057839)
Supplement: Supplementary file 1 [file DataSheet_1.docx]

Supplementary Material

## Supplementary Figures

**1.1**


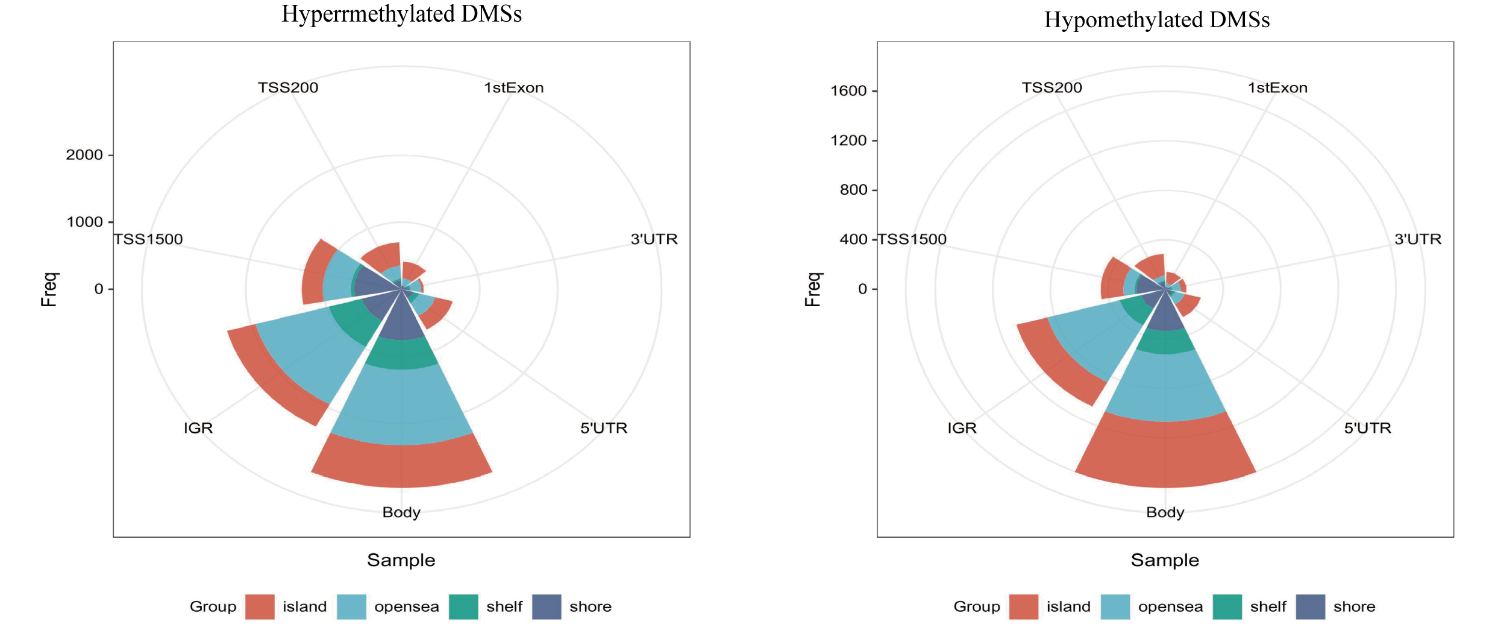


**Supplementary Figure 1: Distribution of hypermethylation DMSs and hypomethylation DMSs in CpG regions and on the genome.**

**1.2**
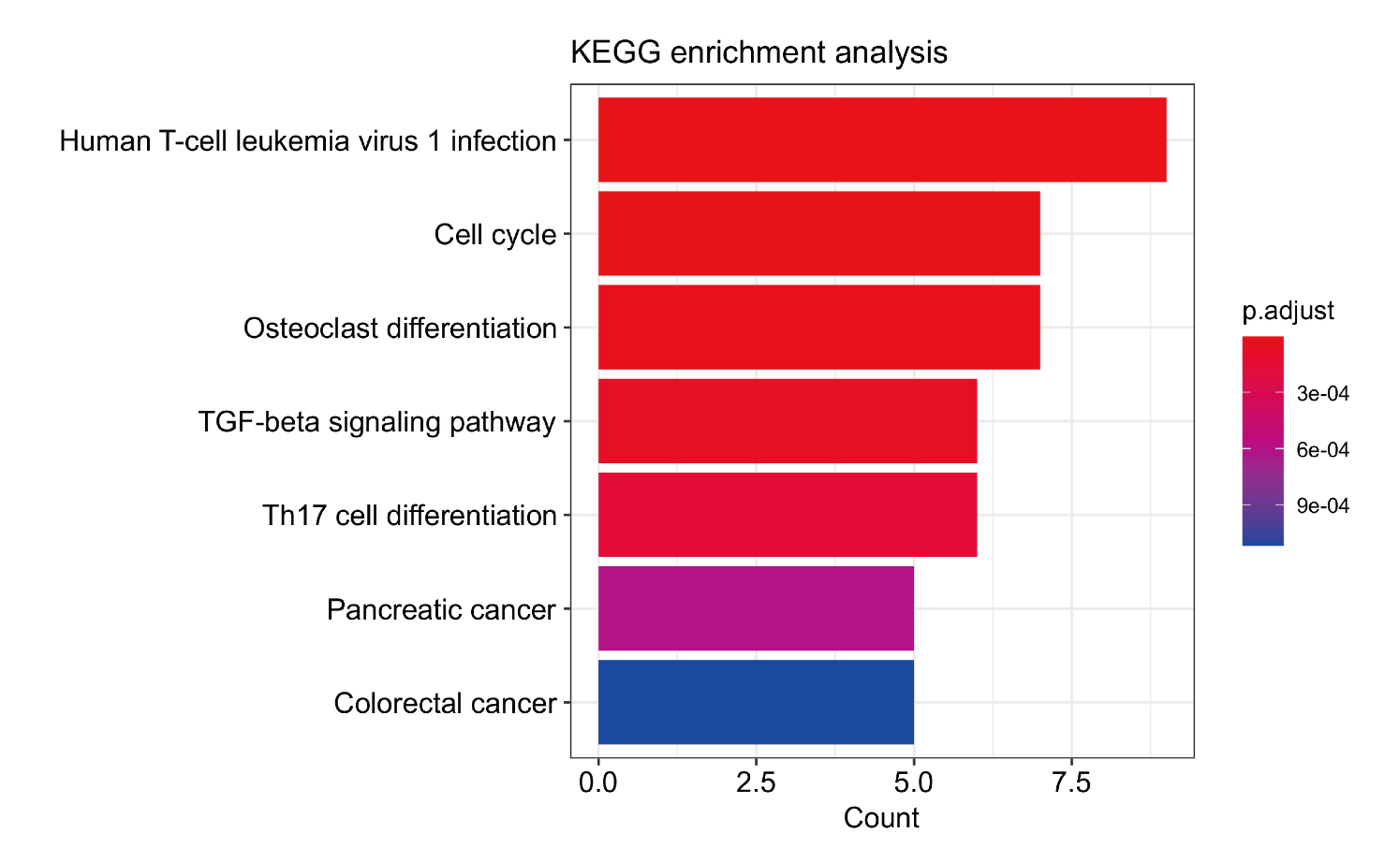


**Supplementary Figure 2: KEGG enrichment analysis of TFs that may be bound to hypermethylated loci**

**1.3**


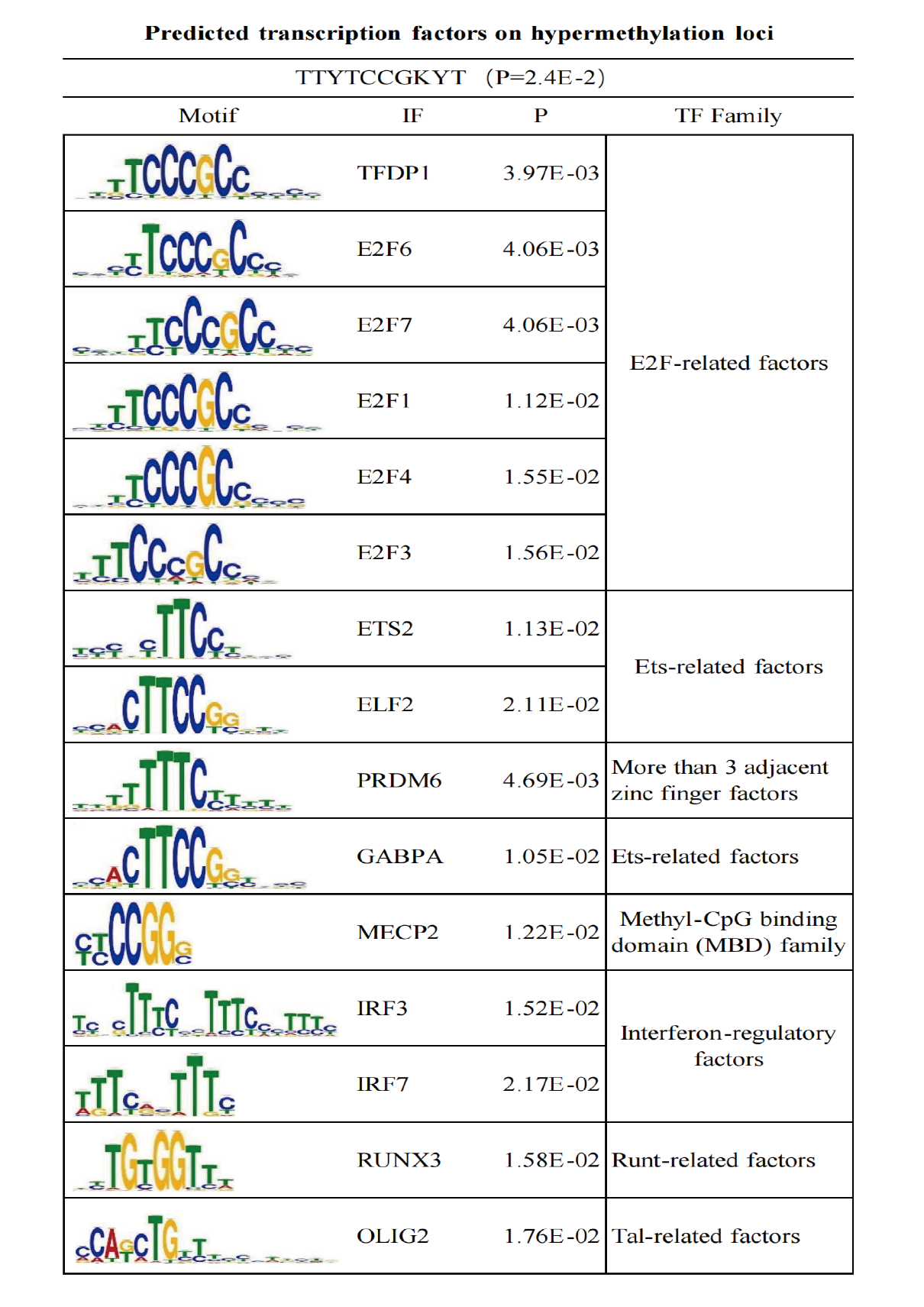


**Supplementary Figure 3:** **Methylation and gene expression correlation analysis**

**1.4**

**
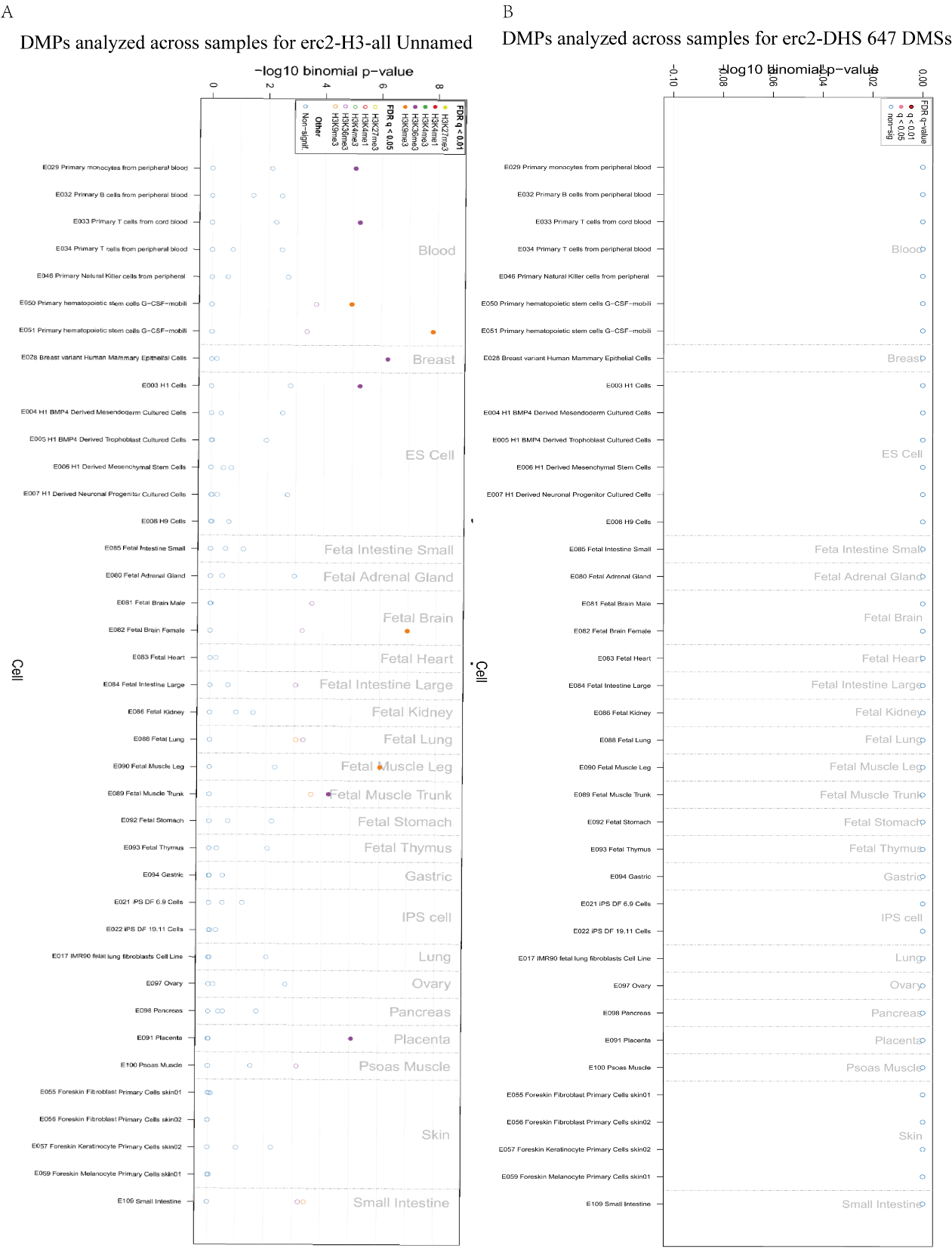
**

**Supplementary Figure 4: Other epigenetic markers Annotation for 647DMSs**

1. **:** The annotation of Five histone mark modifications(A) and DNaseI hotspots(B) for 647 DMSs generated by eFORGE. Statistically significant at P < 0.05.

**1.5**


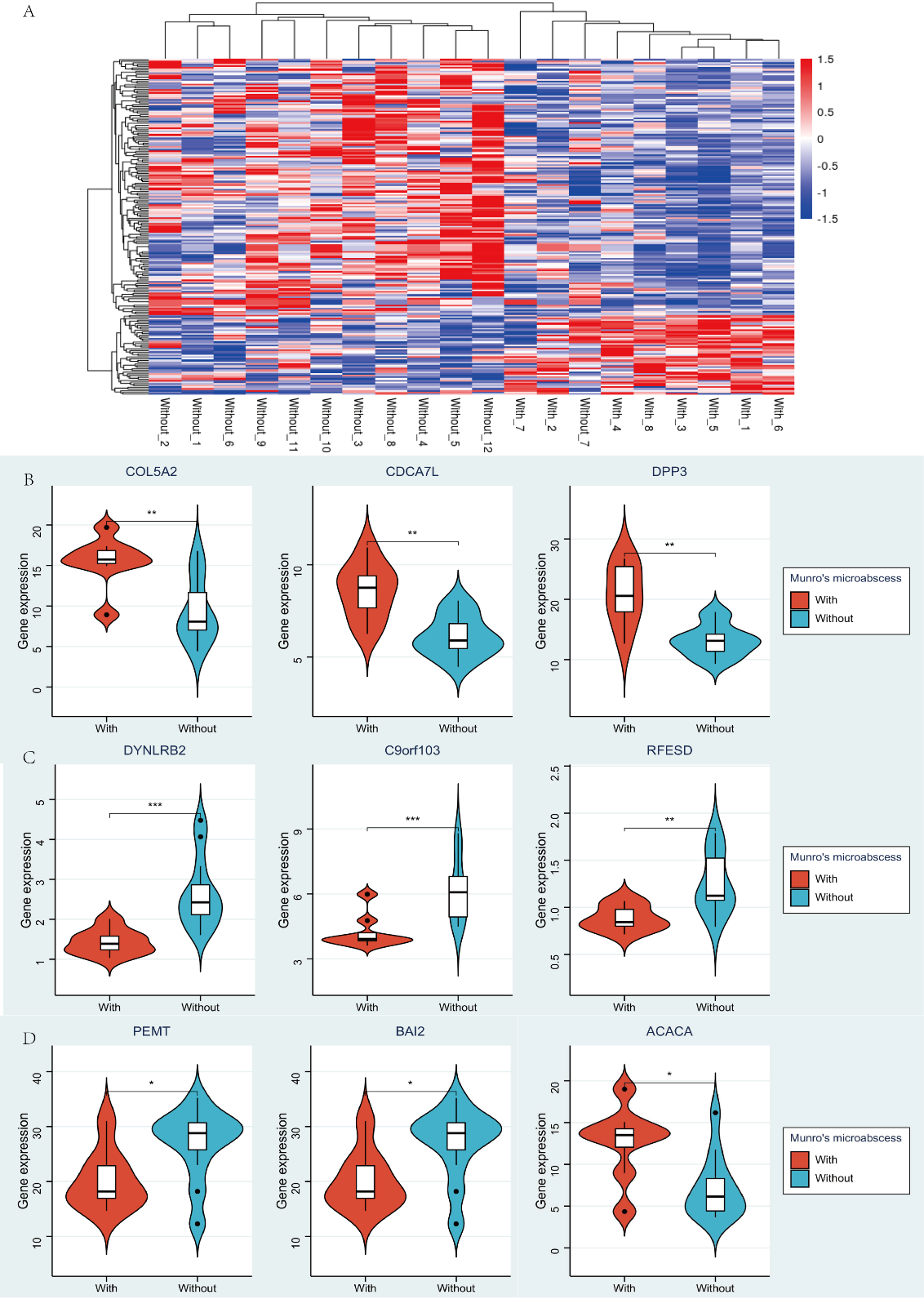


**Supplementary Figure 5: The differential transcription of the absence and presence of Munro's microabscess**

(A): Hierarchical clustering with 201 DEGs shows visibly separate clusters, mostly with and without Munro's microabscess. (B): The top three decreased expression genes for Munro's microabscess compared without. (C): The top three increased expression genes in Munro's microabscess compared without. (D): Differentially expressed genes that can be mapped to differential methylation sites. '***' means P<0.0001; '**' means P<0.001; '*' means P<0.01.

**2. Supplementary Table**

**Supplementary Table 1: The most remarkable hypermethylated and hypomethylated DMGs**

**Supplementary Table 2: GO enrichment for 413 genes mapped by 647 DMSs**

**Supplementary Table 3: Information on 19 genes enriched by Go analysis and their matching probes**

**Supplementary Table 4: Correlation between methylation and gene expression**
